# The Proteome of Human Liver Peroxisomes: Identification of Five New Peroxisomal Constituents by a Label-Free Quantitative Proteomics Survey

**DOI:** 10.1371/journal.pone.0057395

**Published:** 2013-02-27

**Authors:** Thomas Gronemeyer, Sebastian Wiese, Rob Ofman, Christian Bunse, Magdalena Pawlas, Heiko Hayen, Martin Eisenacher, Christian Stephan, Helmut E. Meyer, Hans R. Waterham, Ralf Erdmann, Ronald J. Wanders, Bettina Warscheid

**Affiliations:** 1 Department of Molecular Genetics and Cell Biology, Ulm University, Ulm, Germany; 2 Institut für Biologie II, Funktionelle Proteomik, Fakultät für Biologie and BIOSS Centre for Biological Signalling Studies, Universität Freiburg, Freiburg, Germany; 3 Laboratory of Genetic Metabolic Diseases, Department of Clinical Chemistry and Pediatrics, Academic Medical Center, University of Amsterdam, Amsterdam, The Netherlands; 4 Medizinisches Proteom-Center, Ruhr-Universität Bochum, Bochum, Germany; 5 Leibniz-Institut für Analytische Wissenschaften - ISAS e.V., Dortmund, Germany; 6 Abteilung für Systembiochemie, Medizinische Fakultät, Ruhr-Universität Bochum, Bochum, Germany; Drexel University College of Medicine, United States of America

## Abstract

The peroxisome is a key organelle of low abundance that fulfils various functions essential for human cell metabolism. Severe genetic diseases in humans are caused by defects in peroxisome biogenesis or deficiencies in the function of single peroxisomal proteins. To improve our knowledge of this important cellular structure, we studied for the first time human liver peroxisomes by quantitative proteomics. Peroxisomes were isolated by differential and Nycodenz density gradient centrifugation. A label-free quantitative study of 314 proteins across the density gradient was accomplished using high resolution mass spectrometry. By pairing statistical data evaluation, cDNA cloning and *in vivo* colocalization studies, we report the association of five new proteins with human liver peroxisomes. Among these, isochorismatase domain containing 1 protein points to the existence of a new metabolic pathway and hydroxysteroid dehydrogenase like 2 protein is likely involved in the transport or β-oxidation of fatty acids in human peroxisomes. The detection of alcohol dehydrogenase 1A suggests the presence of an alternative alcohol-oxidizing system in hepatic peroxisomes. In addition, lactate dehydrogenase A and malate dehydrogenase 1 partially associate with human liver peroxisomes and enzyme activity profiles support the idea that NAD^+^ becomes regenerated during fatty acid β-oxidation by alternative shuttling processes in human peroxisomes involving lactate dehydrogenase and/or malate dehydrogenase. Taken together, our data represent a valuable resource for future studies of peroxisome biochemistry that will advance research of human peroxisomes in health and disease.

## Introduction

The peroxisome is a single membrane-surrounded organelle present in virtually all eukaryotic cells. Despite its simple architecture peroxisomes play an essential role in various metabolic pathways including fatty acid (FA) α- and β-oxidation, ether-phospholipid biosynthesis, reactive oxygen metabolism and glyoxylate detoxification [1]. The involvement of peroxisomes in such an array of metabolic functions renders it essential in human beings. Its importance is underscored by the existence of a large variety of inherited diseases directly linked to the absence or impaired functions of human peroxisomes [2], [3]. Despite the fact that much progress has been made with respect to a better understanding of peroxisome biochemistry and related diseases [1], it can be anticipated that additional peroxisomal disorders exist. Detailed knowledge of the proteome of peroxisomes may pave the way for discovering so far unknown disorders caused by the malfunctioning of peroxisomal proteins.

Interestingly, human peroxisomes lack several proteins present in peroxisomes of *Mus musculus* and the yeast *Yarrowia lipolytica* [4] and have so far not been systematically studied by proteomics means. The identification of new constituents of human peroxisomes is highly desirable as it provides the potential of attaining new clues about organelle functions and may shed new light on ill-defined aspects in peroxisome biochemistry such as metabolite transport across the peroxisomal membrane [5].

Mass spectrometry (MS)-based proteomics is a powerful tool to inventory the proteome of subcellular structures [6], [7]. Detailed proteome catalogues of several organelles from different organisms including newly identified *bona fide* constituents have been established by exploiting gradient centrifugation coupled with quantitative MS [8], [9], [10], [11]. The high functional diversity of mammalian peroxisomes accompanied with distinct differences in their molecular composition has been highlighted by Islinger *et al.* [12]. It has to be pointed out here that proteomic studies of mammalian peroxisomes are generally hampered by the limited ability to purify this low abundant and highly dynamic organelle from different tissues. While most MS-based studies focused on the proteomic characterization of rat liver peroxisomes [13], [14], we recently reported a virtually complete inventory of mouse kidney peroxisomes [15].

In this work, we comprehensively studied for the first time the proteome of human liver peroxisomes. To identify new peroxisomal proteins, we accomplished a label-free quantitative MS-based proteomics survey combined with statistical data evaluation, cDNA cloning and colocalization studies by confocal microscopy. We report five new proteins that at least partially locate to human peroxisomes pointing to new metabolic pathways associated with this organelle. Of these, malate dehydrogenase 1 (MDH1) and lactate dehydrogenase A (LDHA) are suggested to assist in the regeneration of NAD^+^ during FA β-oxidation. Furthermore, the label-free strategy outlined here is widely applicable to the accurate and in-depth proteomic characterization of “hard to grasp” organelles from human tissues.

## Materials and Methods

### Clinical Material

Normal liver tissue was collected with informed consent from the patients in compliance with institutional regulations for use of human material. The study goal and design was not for biomarker discovery, verification or validation but for studying the basal protein composition of human liver peroxisomes. A single sample was collected and snap-frozen in liquid nitrogen, and stored at −80°C before further preparation of peroxisomes in the Academic Medical Center, Amsterdam, The Netherlands.

### Enrichment of Peroxisomes from Human Liver, Enzyme Activity Measurements and Immunoblotting

Peroxisomes were isolated from fresh human liver tissue essentially as described previously [16]. This method involves homogenization of human liver in a buffer containing 10 mM morpholinopropanesulphonic acid-NaOH, 250 mM sucrose, 2 mM EDTA, and 0.1% ethanol (final pH value of 7.4) followed by centrifugation of the homogenate at 600×*g* for 10 min at 4°C to produce a post-nuclear supernatant. The postnuclear supernatant was centrifuged at 3,000×*g* for 10 min at 4°C and subsequently the obtained supernatant was centrifuged at 30,000×*g* for 15 min at 4°C to produce a peroxisome-enriched organelle fraction. After resuspension of the organelle fraction in buffer supplemented with 5% Nycodenz, peroxisomes were isolated by Nycodenz equilibrium gradient centrifugation as described [17]. 2 ml fractions were taken from the bottom of the gradient. The collected fractions were assayed using marker enzymes for peroxisomes (catalase), mitochondria (glutamate dehydrogenase), lysosomes (β-hexosaminidase), and microsomes (esterase) as described (see [16] and references therein). In addition, LDH and MDH activities were assayed via the consumption of NADH in the presence of pyruvate and oxaloacetate, respectively. Assays were performed in 50 mM KPi buffer pH 7.4 in the presence of 0.3 mM NADH and 0.1% Triton X-100 at 37°C. Enzymatic reactions were started with the addition of 1 mM pyruvate/oxaloacetate and monitored by 340 nm.

For the analysis of the subcellular location of organellar marker and peroxisomal candidate proteins, 10 µl of Nycodenz gradient fractions 2 to 19 were taken and separated by SDS-PAGE using 5 to 10% gradient gels followed by immunoblotting using antibodies directed against catalase (peroxisomal marker) and voltage-dependent anion channel (VDAC; mitochondrial marker) as well as the two peroxisomal candidate proteins hydroxysteroid dehydrogenase like 2 (HSDL2) and isochorismatase domain containing protein 1 (ISOC1). Anti-HSDL2, anti-ISOC1 (K-14) and anti-VDAC1 were polyclonal antibodies purchased from Abnova Corporation (Taipei City, Taiwan), Santa Cruz Biotechnology (Santa Cruz, CA, USA) and Abcam (Cambridge, UK), respectively. Anti-catalase was the monoclonal antibody 13A10 as described in [18]. Measurements of protein concentration were performed according to Bradford [19] with BSA as standard. For mass spectrometric analyses, 1 ml aliquots of gradient fractions 4 to 12 were taken to prepare pellets by centrifugation at 16,000 g for 10 min. Proteins were resuspended in sample buffer containing 30 mM TrisHCl, 2 M thiourea, and 7 M urea (pH 8.5) to a concentration of 1 µg/µl.

### Analysis of Gradient Fractions by Mass Spectrometry

Protein samples were diluted in 50 mM NH_4_HCO_3_ (pH 7.8) to a final concentration of 0.1 µg/µl. Protein digestion and LC/MS/MS analyses of tryptic peptide mixtures were carried out essentially as described in [15]. Chromatographic separations were performed on a Dionex LC Packings HPLC systems (Dionex LC Packings, Idstein, Germany) coupled to electrospray ionization tandem mass spectrometry (ESI-MS/MS) on a 7-Tesla Finnigan LTQ-FT (Thermo Scientific, Bremen, Germany). The instrument was operated in the data-dependent mode for MS and MS/MS analyses similar to the method described by Olsen *et* *al*. [20]. Survey MS spectra from *m*/*z* 300–1,200 were acquired in the FTICR cell with a mass resolution of 25,000 at *m*/*z* 400 and a target accumulation value of 5,000,000. FTICR single ion monitoring scans (mass window ±5 Da, resolution of 50,000, and target accumulation value of 100,000) were performed on the three most intense ions. Peptide fragmentation was carried out in the linear ion trap by low-energy CID with a target accumulation value of 10,000. Subsequently, precursor ions were dynamically excluded for 45 s. The instrument was operated with a spray voltage of 1.8 kV, an ion transfer tube temperature of 200°C, and for MS/MS, using a normalized collision energy of 35% with an activation *q* of 0.25 for 30 ms. Ion selection thresholds for MS and MS/MS were 1,000 and 500 counts, respectively. For improved proteome coverage, we performed gas phase fractionation in the *m/z* dimension for precursor ion selection in MS/MS scans. Each sample was analyzed thrice with a *m/z* range of 300–1,200 in the MS scan but each time with different overlapping narrow *m/z* ranges covering 400–650, 600–850, and 800–1,200 for the selection of precursor ions in MS/MS scans.

Peaklists of MS/MS spectra acquired on the LTQ-FT (Thermo Scientific) instrument were generated using the software tool Bioworks 3.1 SR 1 with default parameters. For peptide and protein identification, peaklists were correlated with the human International Protein Index (Human IPI V3.41) (www.ebi.ac.uk) database containing 72,155 protein entries using MASCOT (release version 2.2.0) [21]. All searches were performed with tryptic specificity allowing up to two missed cleavages and with oxidation of methionine as variable modification. No fixed modifications were considered. Mass spectra were searched with a mass tolerance of 3 ppm for precursor ions and 0.4 Da for fragment ions. MS/MS spectra were accepted with a minimum MASCOT score of 15. Cut-off scores provided highest number of protein identifications on the basis of one peptide and a false discovery rate (FDR) below 5%. Calculations of the FDR were performed as described [22]. In brief, the exported mass spectra were searched using MASCOT (release version 2.2.0) against a composite database (144,310 entries) consisting of the human IPI and a duplicate of the same database in which the amino acid sequence of each protein entry was randomly shuffled, generated by the publically available Decoy Database Builder (www.medizinisches-proteom-center.de) [23]. Proteins were assembled on the basis of peptide identifications using the ProteinExtractor Tool (version 1.0) in ProteinScape (version 1.3, Bruker Daltonics) and sorted according to their scores. Redundancies in protein entries were removed, i.e. only the protein of lowest molecular weight was reported. Protein isoforms were identified on the basis of at least one unique peptide. The FDR was calculated as the quotient of the number of all proteins identified in the shuffled database and the sum of all protein identifications in both the human IPI database and its shuffled version. Only protein hits up to an accumulated FDR of 5% were considered. Protein annotations were based on entries in the SwissProt (http://www.expasy.org/) database. Lists of peptide and protein identifications as well as profiling data are reported in Tables S1, S2, S3, S4, S5, S6. Protein identification results are accessible in the PRIDE database [24] using the accession key 11791.

### Quantitative Protein Profiling and Statistic

LC/MS runs were analyzed using the software DeCyder MS (version 2.0; GE Healthcare, Uppsala, Sweden). Peptide peaks were detected with an average peak width of 0.5 min and matched with a mass accuracy of at least 0.01 Da and a maximum time window of 4 min. Next, the abundance of individual peptides in the respective gradient fraction was calculated by peak integration (Table S6). Data processing was manually inspected and overlapping peaks were discarded. MS/MS spectra correlated to peptide peaks were searched against the Human IPI V3.30 database using the MASCOT™ algorithm using the same parameters described above. The resulting peptide identifications were imported to Decyder MS and the respective FDR was calculated following the concept described [22]. For quantitative protein profiling, only proteins identified by multiple peptides with a total MASCOT score of 50 or a single peptide with a MASCOT score greater 39, equivalent to a peptide FDR of 1%, were considered.

Peptide profiles were calculated based on the quantitative analysis of MS data of three replicates (Table S6). Each peptide profile was then normalized by setting the highest intensity to one. For the establishment of protein profiles, peptide profiles were averaged and a standard deviation was calculated (Table S4). For statistical analysis, the mean profile of 46 known peroxisomal proteins with measured peptide intensities in at least two different gradient fractions was calculated. Next, the Euclidian distance (Ρ) between this mean profile and all individual protein correlation profiles was calculated for fractions 4 to 12. Proteins were sorted according to their Ρ value. A Receiver Operating Characteristics (ROC) curve [25] with an area under curve (AUC) of 0.957 (data not shown) was calculated, indicating the accuracy of this method. Non-peroxisomal proteins were required to exhibit (i) a specificity of ≥96.5%, equivalent to a Ρ value of 0.9, and (ii) two quantified peptides to be considered as potential peroxisomal candidate protein.

### Cell Culture and Confocal Microscopy

Huh7 [28] cells, which were a kind gift of L. Rudolph (Ulm University, Germany), were cultivated in Dulbecco’s modified Eagle medium (DMEM; Gibco/Invitrogen, Karlsruhe, Germany) supplemented with 10% fetal calf serum (FCS; Gibco). One day before transfection, cells were seeded on glass coverslips to a density of 250,000 cells/ml. The medium was exchanged by DMEM/5% FCS and transfection of cells using the calcium phosphate method was essentially performed as described [26]. Peroxisomal candidate proteins were expressed as DsRed fusion proteins under the control of a cytomegalovirus (CMV) promoter. A list of primers used in this study and details on the cloning procedure are given in Table S7 and the Text S1. We utilized EGFP fused to the peroxisomal targeting signal (PTS) 1 sequence SKL as peroxisomal marker. EGFP-SKL constructs were used previously to identify and characterize new peroxisome assembly mutants in yeast [27] and for colocalization studies in BGL69 cells [14]. The extinction and emission spectra of DsRed and GFP do not interfere [28].

Routinely, 3.0 µg of plasmid and 0.1 µg of pEGFP-Peroxi plasmid (BD Biosciences/Clontech) as peroxisomal marker, GFP-Mito plasmid (BD-Pharmingen, Heidelberg, Germany) as mitochondrial marker and LAMP1-mGFP plasmid as marker for late endocytic organelles (i.e. late endosomes and lysosomes) [29] were cotransfected. After 4 h of transfection, the supernatant was aspirated and the cells were incubated with 10% glycerol in DPBS (Gibco) for 1 min. Cells were grown for further 24 h in DMEM/10% FCS, then fixed for 15 min with 3% paraformaldehyde in DPBS, washed with DPBS and finally mounted on glass slides using VectaShield mounting medium (Vector Laboratories, Burlingame, CA). Analyses were performed on an Observer SD confocal microscope (Zeiss, Göttingen, Germany) equipped with a Plan-Apochromat 63x (lens aperture 1.4) objective, an AxioCam MRm camera and 488 nm and 547 nm diode lasers. Image acquisition and processing was performed with the ZEN blue software (version 1.0; Zeiss).

## Results

### MS-based Proteomics Profiling of Human Liver Peroxisomes

**Figure 1:**
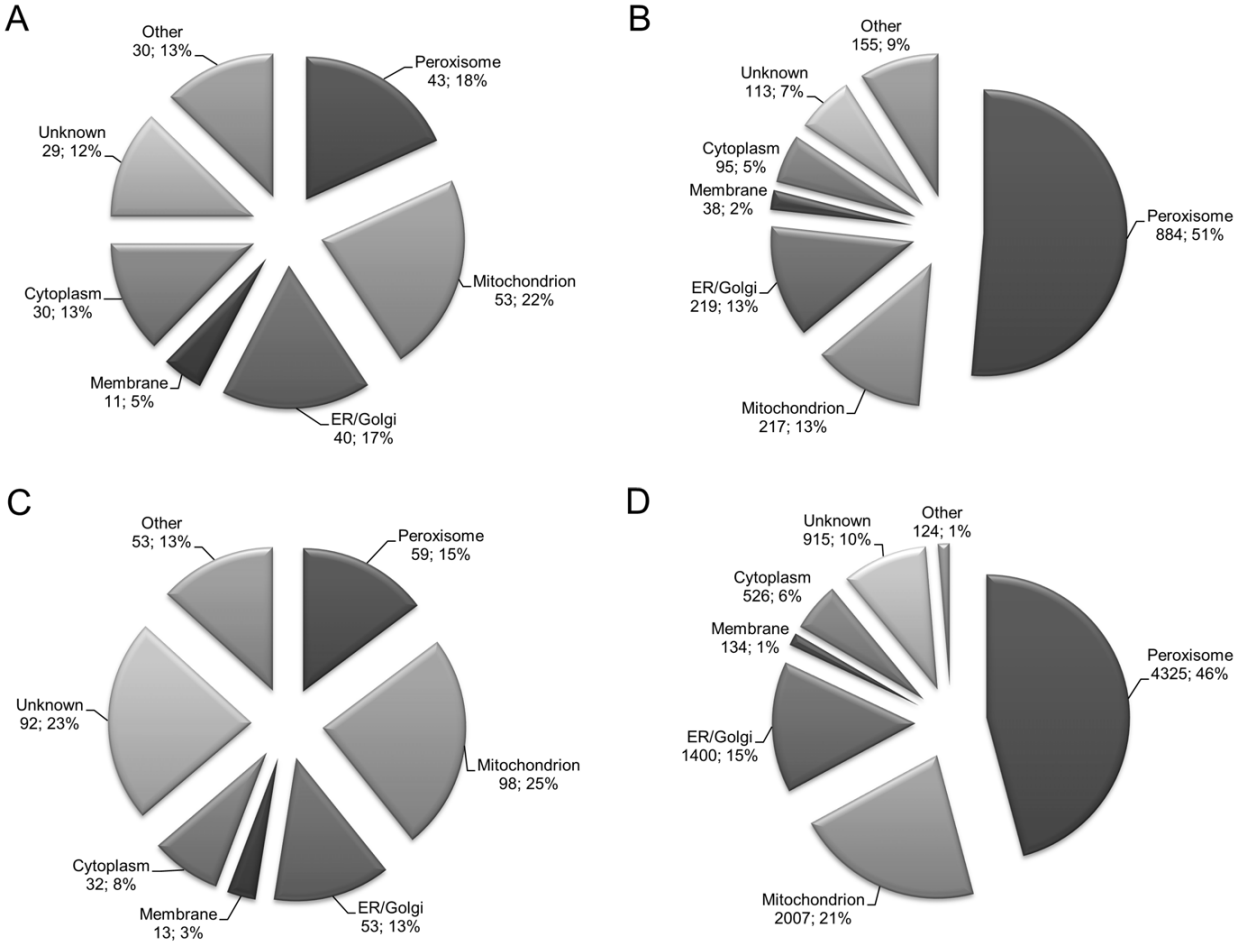
Mass spectrometry-based proteomics analysis of human liver peroxisomes. Peroxisomes were isolated by differential and Nycodenz density gradient centrifugation and subjected to triplicate LC/MS/MS analyses for protein identification. Subcellular distribution of 263 and 400 proteins according to identification numbers (A, C) and spectral counts (B, D) that were detected in the peroxisomal peak fraction 6 and in nine consecutive gradient fractions adjacent to the peroxisomal peak fraction, respectively.

The success of organellar proteomics endeavours generally relies on the adequate isolation of the organelle of interest by biochemical means. To isolate peroxisomes from human liver, we prepared a postnuclear supernatant by differential centrifugation that was further subjected to Nycodenz equilibrium gradient centrifugation according to Ofman *et* *al.* [16]. Isolation of human liver peroxisomes was studied by enzyme activity measurements of selected organelle marker proteins. Peroxisomes were found to be enriched in the gradient fractions of higher density with fraction 6 as peroxisomal peak fraction. We achieved a good separation of peroxisomes from mitochondria and both lysosomes and microsomes that showed highest enrichment in the lower-density part of the gradient (fraction 14 and fraction 16, respectively; Fig. S1). To further evaluate the molecular composition of the peroxisomal peak fraction 6 in an unbiased approach, we performed triplicate LC/MS/MS analyses on a high resolution LTQ-FTMS instrument. Deep MS-based peptide sequencing was achieved by combining single ion monitoring and gas-phase fractionation as described [15], resulting in the identification of a total of 236 proteins (Table S1). Analysis of the subcellular localization of identified proteins revealed that peroxisomal proteins represent 18% (43 proteins), while the majority originated from mitochondria (22%, 53 proteins), the endoplasmic reticulum/Golgi (17%, 40 proteins), cytoplasm (13%, 30 proteins), membranes (5%, 11 proteins), other compartments (13%, 30 proteins), or were of unknown localization (12%, 29 proteins) as depicted in Fig. 1A. To provide an estimate for the enrichment of peroxisomes in fraction 6, relative protein quantification based on spectral counts (SC) [30] was performed that resulted in an abundance ratio of 51% (884 SC) for the total of peroxisomal proteins to non-peroxisomal proteins (Fig. 1B and Table S1). Furthermore, the peroxisomal multifunctional enzyme type 2 (173 SC), enoyl coenzyme A (83 SC), catalase (74 SC), isoform SCPx of non-specific lipid-transfer protein (59 SC), hydroxyl acid oxidase (53 SC), serine-pyruvate aminotransferase (better known as alanine glyoxylate aminotransferase, AGT) (88 SC) and acyl-coenzyme A oxidase (31 SC), all known to reside in peroxisomes, were the most abundant proteins detected in the peroxisomal peak fraction 6 (Table S1). Based on these data, we conclude that peroxisomes were successfully isolated from human liver; however, the concurrent presence of a high number of co-purified proteins from other cellular compartments generally hampers the accurate proteomic characterization of human liver peroxisomes and may further impede the identification of low abundant and/or so far unknown constituents.

To address this issue, we set out to perform a label-free quantitative proteomics survey of human liver peroxisomes following the concept of protein correlation profiling introduced by Andersen *et* *al.* [8]. To this end, the proteome composition of each of the nine consecutive density gradient fractions 4 to 12 adjacent to the peroxisomal peak fraction 6 was studied in triplicate analyses by LC/MS/MS. Joint evaluation of all the acquired MS/MS data led to the identification of a total of 400 proteins (Table S2). Of these, 59 (15%) referred to known peroxisomal proteins. The remaining proteins derived from mitochondria (25%, 98 proteins), the endoplasmic reticulum/Golgi (13%, 53 proteins), the cytoplasm (8%, 32 proteins), membranes (3%, 13 proteins), other compartments (13%, 53 proteins) or were of unknown localizations (23%, 92 proteins) (Fig. 1C). Based on the quantitative evaluation of proteins identified across gradient fractions 4 to 12 by spectral counting, we estimated that peroxisomal proteins (4325 SC) contributed to approximately half of the total abundance of all proteins in the gradient (Fig. 1D). We generally found a similar subcellular distribution of proteins identified in the peroxisomal peak fraction 6 and in fractions 4 to 12. The slight relative percentage increase of mitochondrial proteins identified in the nine consecutive fractions can likely be attributed to the distribution of mitochondria in the density gradient, which showed an increasing abundance starting with fraction 10 (Fig. S1). As a benefit, LC/MS/MS analyses of gradient fractions adjacent to the peroxisomal peak fraction 6 resulted in both higher spectral count numbers and identification scores of peroxisomal proteins and resulted in the identification of further 16 peroxisomal proteins comprising, for example, GNPAT, ACOT1, PEX1 and PEX5 (Table S2).

The protein inventory of human liver peroxisomes accomplished in this work comprised virtually all constituents of the peroxisomal FA β- and α-oxidation system and reactive oxygen metabolism as well as 15 proteins known to be associated with peroxisomal membranes as summarized in Table S3. Notably, we identified PMP52, a peroxisomal membrane protein of unknown function, the acyl-coenzyme A dehydrogenase ACAD11, the dual localized protein MOSC2, the acyl-coenzyme A binding domain containing protein ACBD5, the AAA domain containing protein ATAD1 and the fatty aldehyde dehydrogenase variant form ALDH3A2 in human liver peroxisomes. Orthologs of all these proteins have recently been reported as new constituents of rat liver and/or mouse kidney peroxisomes [14], [15].

Beyond the mere identification of proteins in density gradient fractions, detailed quantitative assessment of the acquired LC/MS/MS data provides the unique potential to reliably discriminate between peroxisomal proteins and co-purifying contaminants and, thus, to pinpoint new candidate proteins of human liver peroxisomes without beforehand information. MS-based protein profiles are typically established by calculating the normalized peptide abundance of each protein and plotting the averaged values against the respective gradient fractions. Protein profiles derived from the same organelle are expected to correlate to a high degree and resemble distributions of well-accepted organelle marker proteins in the gradient [8], [10], [11]. Protein profiling by quantitative MS thus enables to conduct an unbiased study of proteins located in a distinct organelle.

### Identification of New Candidate Proteins of Human Liver Peroxisomes by Statistical Evaluation

**Figure 2:**
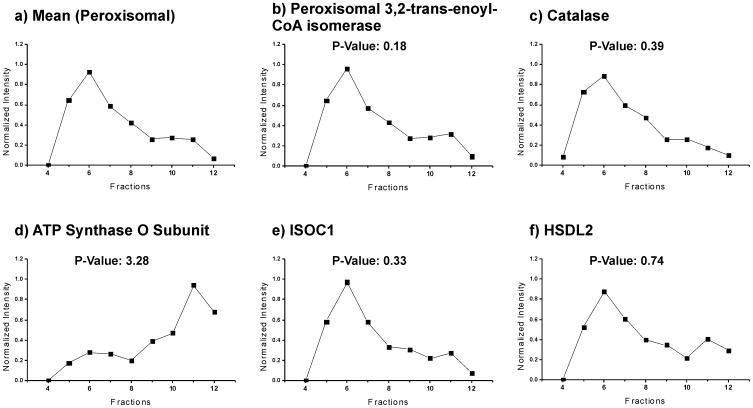
Quantitative protein profiling to identify new candidate proteins of human liver peroxisomes. Protein profiles were established as an average of normalized peptide abundances and correlated to the mean peroxisomal profile by statistical analysis using the χ^2^-method. (A) Mean peroxisomal profile. (B-D) Profiles of peroxisomal 3,2-trans-enoyl-CoA isomerase and catalase as well as the mitochondrial O subunit of ATP synthase. (E, F) Profiles of ISOC1 and HSDL2 identified as new candidate proteins of human liver peroxisomes.

For label-free quantitative analyses, all acquired MS data were processed with the software DeCyder MS as described in Methods. Using this approach, we quantitatively followed more than 2800 unique peptides across the nine consecutive gradient fractions 4 to 12 (Table S6). Based on these data, we quantified 314 proteins for which the respective abundance profiles were calculated (Tables S4 and S5). The profile of each protein was then compared to the mean peroxisomal protein profile using a supervised statistical approach, the χ^2^-method. For each protein a “goodness of fit” was defined that is expressed by the Euclidian distance Ρ (Table S4). Figure 2 depicts (a) the mean peroxisomal profile as well as the profiles of (b) peroxisomal 3,2-trans-enoyl-CoA isomerase, (c) catalase and (d) the O subunit of ATP synthase as resident proteins of peroxisomes (b and c) and mitochondria (d), respectively. Abundance profiles of the newly identified peroxisomal candidate proteins isochorismatase domain containing protein 1 (ISOC1) (e) and hydroxysteroid dehydrogenase like 2 (HSDL2) (f) correlated well with the mean peroxisomal profile as illustrated by their Ρ-values of 0.33 and 0.74, respectively. From a total of six proteins with Ρ values ≤0.9, equivalent to a specificity of 96.5%, and ≥2 peptides quantified, we selected five proteins to further study their cellular localization. For the candidate protein HSDL2 both function and localization are so far unknown. Further three candidate proteins, namely LDHA, MDH1 and alcohol dehydrogenase 1A (ADH1A), were assigned to other subcellular compartments than peroxisomes. Interestingly, one candidate protein, ISOC1, is linked to peroxisomes by gene ontology annotation. As a result of sequence searches of candidate proteins using PSORT [31], a PTS1 (ARL, C-terminal) and both a PTS1 (SKV, C-terminal) and PTS2 (RLVPLQIQL, N-terminal) were predicted for HSDL2 and ISOC1, respectively (Table 1). Using the PTS1 predictor [32], putative PTS1 sequences were equally found in ISOC1 and HSDL2 with scores of 3.02 and 4.97, respectively.

**Table 1:** New candidate proteins of human liver peroxisomes studied by confocal microscopy.

Name	Gene	IPI accession no.	Information on functional domains, transmembrane domains (TMDs), and further comments	PTS predicted by PSORT	Localization listed by UniProt	DsRed fused to the N–/C-terminus of the protein	Localization by confocal microscopy	Ρ-value
Isochorismatase domain-containing protein 1	*ISOC1*	00304082	No annotation. Contains isochorismatase domain.	SKV (PTS1);RLVPLQIQL (PTS2)	GO: peroxisomal	C-terminus	peroxisomal	0.33
						N-terminus	peroxisomal & cytoplasmic	
Hydroxysteroid dehydrogenase like 2	*HSDL2*	00414384	Contains an SCP2 domain.	ARL (PTS1)	GO: peroxisomal,mitochondrial	C-terminus	peroxisomal & mitochondrial	0.74
						N-terminus	peroxisomal & cytoplamic	
L-lactate dehydrogenase A	*LDHA*	00217966	Fermentation; pyruvate fermentation to lactate; lactate from pyruvate. A peroxisomal lactate shuttle mechanism has been postulated	none	cytoplasmic	C-terminus	peroxisomal & cytoplasmic	0.52
Alcohol dehydrogenase 1A	*ADH1A*	00218896	Zinc-dependent alcohol dehydrogenase	none	cytoplasmic	C-terminus	peroxisomal & cytoplasmic	0.89
Malate dehydrogenase 1	*MDH1*	00291005	Protein of the citric acid cycle.	none	cytoplasmic	C-terminus	peroxisomal & cytoplasmic	0.88

**Figure 3:**
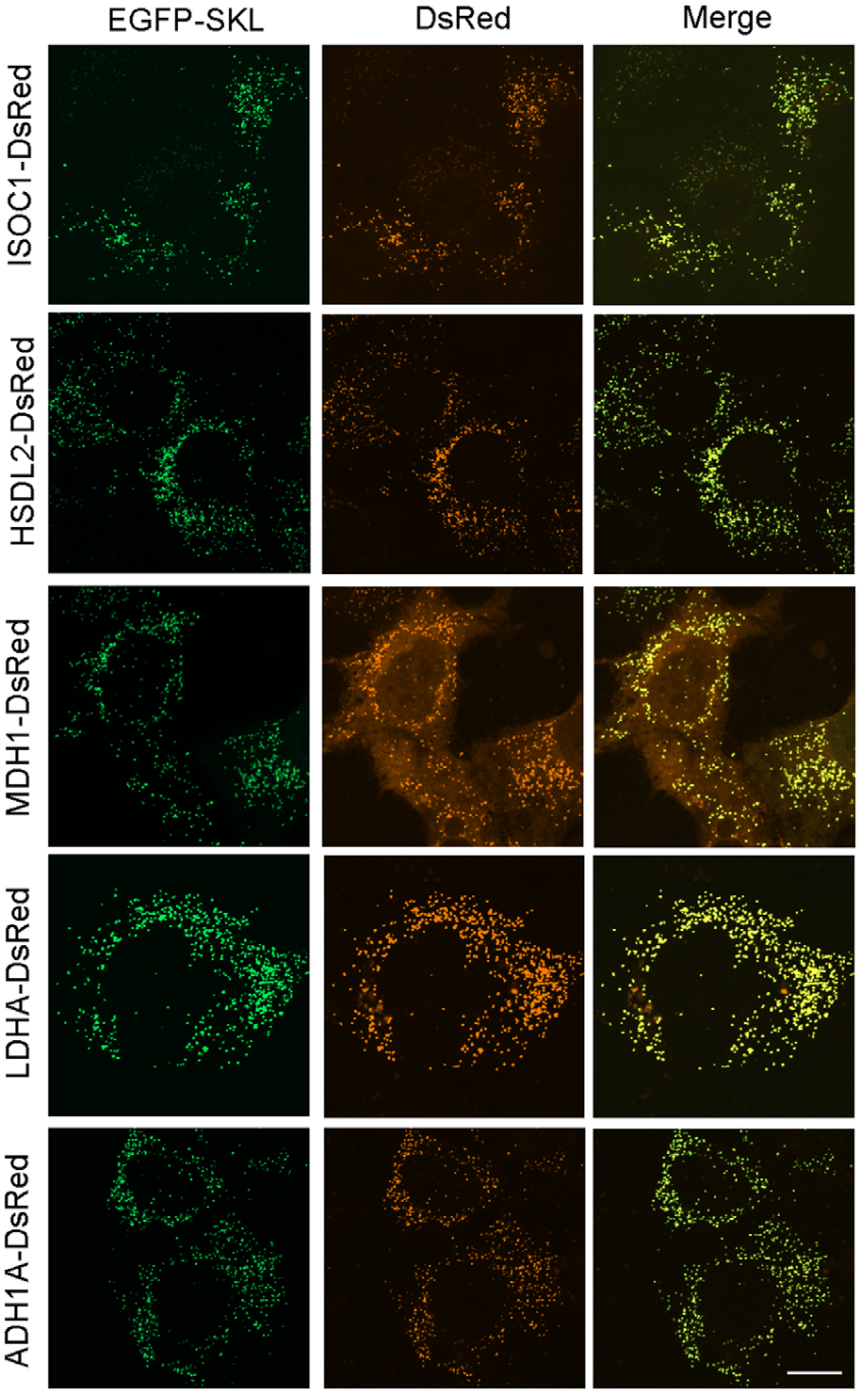
Localization of five new candidate proteins to human peroxisomes. Huh7 cells were transfected with plasmids for expression of DsRed fusion proteins of ISOC1, HSDL2, MDH1, LDHA and ADH1A as well as EGFP-SKL as peroxisomal marker. Images from the left to right: EGFP-SKL (green), candidate fusion protein (red), merge. Images were assembled from z-projections; the scale bar represents 20 µm.

Human liver peroxisomes were enriched by differential and Nycodenz gradient centrifugation (see Fig. S1). Ten consecutive gradient fractions were quantitatively analyzed by high resolution MS to establish abundance profiles of 314 proteins (Fig. 2, Table S4). Using a supervised statistical approach, the χ^2^-method, new candidates of human liver peroxisomes were identified with an Euclidian distance (Ρ-value) of ≤1.02. All candidate proteins were shown to associate with human peroxisomes by colocalization studies using confocal microscopy in Huh7 (Fig. 3, Fig. S2 and S6). Sequences of candidate proteins were analyzed for potential peroxisomal targeting signals (PTS) 1 and 2 using both PSORT [31] and the PTS1 predictor [35].

### Association of New Candidate Proteins with Peroxisomes *in vivo*

To confirm the association of candidate proteins with human liver peroxisomes, we conducted colocalization studies in the human hepatoma cell line Huh7 by confocal microscopy. Peroxisomal candidate proteins were expressed in Huh7 cells as DsRed fusion proteins under the control of a CMV promoter. We used EGFP fused to the PTS1 SKL as peroxisomal marker. If a protein localizes to peroxisomes, its expression pattern should correspond to the punctate pattern of EGFP-SKL. Peroxisomal candidate proteins were generally fused at their respective C-terminus to the N-terminus of DsRed. In case of ISOC1 and HSDL2, each of which containing a predicted C-terminal PTS1, we also studied the N-terminally tagged versions. We further studied the localization of such DsRed fusion proteins to mitochondria using EGFP-Mito as well as late endocytic organelles (i.e. late endosomes and lysosomes) using the marker LAMP1-mGFP, a non-dimerizing mutant variant of human LAMP1 (lysosome-associated membrane protein 1) fused at its cytoplasmic tail to GFP [29]. As a result of *in* *vivo* colocalization studies, we confirmed that ISOC1, HSDL2, MDH1, LDHA and ADH1A associate with human peroxisomes (Fig. 3 and Fig. S2). For all the proteins a punctuate pattern was observed that corresponded well to the fluorescent pattern of the peroxisomal marker EGFP-SKL. In agreement with the known cytosolic location of MDH1, LDHA and ADH1A, we found a certain amount of the respective fusion proteins in the cytosol. As controls, we demonstrate that (i) the known peroxisomal protein PMP52 when fused at its C-terminus to DsRed colocalizes with EGFP-SKL and (ii) the peroxisomal (EGFP-SKL) and the mitochondrial (DsRed-Mito) marker did not colocalize when transiently expressed in Huh7 cells (Fig. S3). In addition, late endocytic organelles exhibiting a similar subcellular localization pattern as peroxisomes were detected with the marker LAMP1-mGFP, but none of the new peroxisome-associated proteins colocalized with these organelles (Fig. S4). Moreover, for none of the proteins an association with mitochondria was found with the exception of HSDL2 displaying a dual localization (Fig. S5). It is of interest to note that HSDL2 when fused at its respective N-terminus to DsRed did not colocalize with the mitochondrial marker EGFP-Mito, but both N-terminally tagged HSDL2 and ISOC1 colocalized with the peroxisomal marker EGFP-SKL. A fraction of each fusion protein was also observed in the cytosol (Fig. S6).

**Figure 4:**
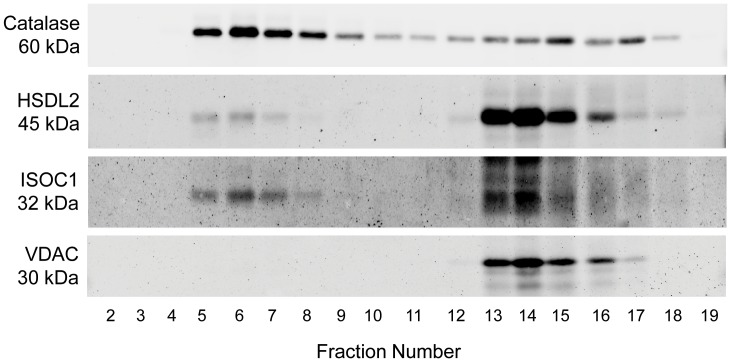
ISOC1 and HSDL2 partially colocalize with human liver peroxisomes. Nycodenz gradient fractions of human liver were subjected to SDS-PAGE followed by immunoblot analysis using antibodies against the peroxisomal candidate proteins HSDL2 and ISOC1 as well as catalase (peroxisomal marker) and the voltage-dependent anion channel VDAC (mitochondrial marker). In addition to its partial localization to peroxisomes, HSDL2 was found to localize to mitochondria.

To corroborate subcellular localization data for ISOC1 and HSDL2 obtained by MS-based protein profiling and confocal microscopy, we followed the distribution of the two proteins as well as catalase (peroxisomal marker) and the voltage-dependent anion channel VDAC (mitochondrial marker) along the human liver Nycodenz density gradient by immunoblot analysis (Fig. 4). As indicated by the signals for catalase and VDAC, peroxisomes and mitochondria were separated in the density gradient and immunoblot data were consistent with enzyme activity profiles of catalase and the mitochondrial marker protein glutamate dehydrogenase (GlutDH) (Fig. S1). Peroxisomes were found to be enriched in the high density gradient fractions 5 to 9. In contrast, mitochondria were absent in these fractions and showed highest enrichment in the lower density fractions 13 to 16 (Fig. 4, Fig. S1). For HSDL2, we observed a small but distinct portion of the endogenous protein in fractions 5 to 8, whereas the majority of the protein was detected in fractions 13 to 16. Thus, our data confirm that HSDL2 exhibits a dual localization. We also detected ISOC1 in high density fractions 5 to 8, while signals in the lower density fractions 13 to 17 most likely represent background staining resulting from a certain degree of cross-reactivity between the polyclonal anti-ISOC1 antibody and abundant proteins present in these fractions. To conclude, our data confirm the association of ISOC1 with human peroxisomes, while HSDL2 is located to both peroxisomes and mitochondria in human cells.

Interestingly, our quantitative MS and confocal microscopy data further point to the association of LDHA and MDH1 with human liver peroxisomes. Both enzymes belong to the LDH/MDH superfamily and are known to be located in the cytoplasm in human cells. To provide additional supporting data for a possible function of these oxidoreductases in human liver peroxisomes, we measured the activities of LDH and MDH in the human liver Nycodenz density gradient (Fig. S1). Both enzymes showed activity profiles with local maxima in the peroxisomal peak fraction 6. Activities of MDH and LDH increased again starting from fraction 11 with main activities in lower-density fractions of the gradient. The latter finding can likely be attributed to the concurrent presence of different isoforms of LDH and MDH. For example, activity of LDH in mitochondria has been reported before [33] and since MDH1 and its mitochondrial isoform MHD2 exhibit the same substrate specificity, the acquired enzyme activity profile presumably reflects the activities of both isoforms. Altogether, our data support the idea that small portions of LDHA and MHD1 are indeed associated with human peroxisomes. Thus, it will be of great interest to further investigate whether these enzymes are indeed transported across the peroxisomal membrane in future work.

## Discussion

We followed a quantitative organellar proteomics approach to study for the first time the proteome of human liver peroxisomes. Differential and Nycodenz density gradient centrifugation of a postnuclear supernatant of human liver enabled to isolate peroxisomes in the high density part of the gradient for subsequent peptide sequencing analyses by high resolution LC/MS/MS (Fig. S1). Proteomic analysis of the peroxisomal peak fraction 6 resulted in the identification of 43 known peroxisomal proteins, though numerous proteins from other subcellular compartments such as the mitochondrion and endoplasmic reticulum were also present (Fig. 1A; Table S1). Nonetheless, successful isolation of peroxisomes from human liver was confirmed by the findings that (i) peroxisomal proteins contributed to approx. 50% of all spectral counts (Fig. 1B) and (ii) key enzymes of peroxisomes were of highest abundance (Table S1). Yet, the presence of a relatively large fraction of copurified non-peroxisomal proteins was expected to possibly hamper the identification of low abundant peroxisomal proteins. To address this issue, we thoroughly analyzed consecutive gradient fractions adjacent to the peroxisomal peak fraction allowing us to (i) accumulate further peptide-based evidence of peroxisomal proteins and (ii) accurately follow the distribution of proteins across the density gradient by label-free quantitative MS. To provide highest sensitivity for peroxisomes, we focused in our analysis on the fractions 4 to 12 in the dense part of the gradient. This approach led to the identification of a total of 59 known peroxisomal proteins with a high number of spectral counts (Fig. 1C and 1D; Table S2). The protein inventory of human liver peroxisomes reported here comprises virtually all proteins with essential functions in peroxisome biochemistry and biogenesis (Table S3). Interestingly, the nudix hydrolase RP2p remained unidentified in human liver as well as mouse/rat liver peroxisomes, whereas it has been reported to be highly expressed in mouse kidney [13], [15]. Moreover, ZADH2 has readily been identified in rat/mouse peroxisomes [14], [15], while it could not be detected in human liver peroxisomes in this work. Despite these differences in the protein composition of peroxisomes from different tissues and/or species, we identified human orthologues of numerous peroxisomal proteins such as PMP52, ACAD11, ACBD5, ATAD1, ALDH3A2 and MOSC2 known to be expressed in rodents [13], [14], [15]. Based on these findings, we considered our quantitative protein profiling approach to be well suited for the discovery of new candidate proteins of human liver peroxisomes. Label-free high resolution LC/MS/MS analyses allowed to infer abundance profiles of 314 proteins from more than 2800 peptides using DeCyder MS and MASCOT for quantitative data evaluation (Tables S4, S5 and S6, Fig. 2). Applying stringent criteria (Ρ value ≤0.89, ≥2 peptides identified and quantified), ISOC1, HSDL2, MDH1, LDHA and ADH1A were selected as new peroxisomal candidate proteins and further subjected to colocalization studies by confocal microscopy as summarized in Table 1. The *in* *vivo* colocalization studies revealed for all the candidate proteins a punctuate pattern that corresponded well to the fluorescence pattern of the peroxisomal marker EGFP-SKL and, thus, provided further evidence for their association with human peroxisomes (Fig. 3, Fig. S2 and S6). None of the proteins colocalized with late endocytic organelles (Fig. S4) and with the exception of HSDL2, for none of the proteins an association with mitochondria was observed (Fig. S5). In addition, immunoblot analyses of human liver Nycodenz density gradient fractions confirmed the dual localization of HSDL2 and the association of ISOC1 with human peroxisomes (Fig. 4).

### New Peroxisome-associated Proteins of Unknown Functions

We demonstrated the association of ISOC1 and HSDL2, two proteins of unknown function, with human liver peroxisomes by independent means. ISOC1 contains an isochorismatase domain and may be involved in the hydrolysis of ether bonds. Isochorismatase catalyzes the conversion of isochorismate to 2,3-dihydroxybenzoate and pyruvate and is part of the shikimic acid pathway in microorganisms [34]. However, there are several isochorismatase domain-containing proteins in mammals. Furthermore, ISOC1 is widely conserved throughout several species with homologues in, for instance, *Mus musculus* and *Bos taurus*. ISOC1 was previously identified in peroxisomal preparations of rat liver [14]. Two PTS were predicted for ISOC1; a putative PTS1 (SKV) located at the C-terminus and a putative PTS2 (RLVPLQIQL) in the N-terminal region of the protein. Interestingly, ISOC1 when fused with its C- or N-terminus to DsRed colocalized with peroxisomes in Huh7 cells (Fig. 3, Fig. S2 and S6). A fraction of N-terminally tagged protein was also found in the cytosol, which may represent an artifact of overexpression but may also indicate that an accessible PTS2 is required for effective localization of ISOC1 to peroxisomes. Nevertheless, the data generally allow for the assumption that both peroxisomal targeting signals may be functional. Thus, it will be of interest to elucidate the mechanism underlying the targeting of ISOC1 to human peroxisomes and, moreover, its yet unknown function in the metabolism of human cells.

HSDL2 is the product of one of the five human sterol carrier protein 2 (SCP2) domain encoding genes [35], thus pointing to its potential involvement in the transport and/or metabolism of fatty acids. Interestingly, HSDL2 has recently been identified in mouse kidney peroxisomes [36] and, moreover, rat SCP2 protein has been reported to reside in both peroxisomes and mitochondria [37]. The substrate of HSDL2 remains yet to be identified but since the protein exhibits a SCP domain, a putative role in fatty acid metabolism was suggested [38]. Interestingly, HSDL2 has been shown to play a central role in a genetic network regulating the accumulation of cholesterol-esters [39].

In HeLa cells, N- and C-terminally tagged versions of human HSDL2 have been reported to localize to peroxisomes and mitochondria, respectively [38]. In this work, we showed that endogenous HSDL2 localizes to both peroxisomes and mitochondria of human liver (Fig. 4). We further observed that both C- and N-terminally tagged versions of HSDL2 localized to peroxisomes, while, as expected, localization to mitochondria was blocked when HSDL2 was fused with its N-terminus to DsRed in Huh7 cells (Fig. 3, Fig. S2 and S5). Our data therefore consistently point to a dual localization of human HSDL2 *in* *vivo*. The enzyme may likely be routed to peroxisomes via its putative PTS1 (ARL, C-terminal); however, the existence of a PTS1-independent import pathway cannot be excluded and needs further investigation.

### New Proteins Associated with Human Liver Peroxisomes with Functional Annotations

We identified three distinct oxidoreductases, ADH1A, LDHA and MDH1, as new peroxisomal candidate proteins in our quantitative proteomics screen. Additional evidence for the partial association of these metabolic enzymes with human peroxisomes resulted from colocalization studies in Huh7 cells by confocal microscopy (Fig. 3 and Fig. S2). Our findings thus open up the possibility of the existence of new functional roles of these proteins in the biochemistry of peroxisomes as discussed below.

### Identification of Alcohol Dehydrogenase 1A Points to a New Alcohol-oxidizing System Associated with Hepatic Peroxisomes

In human liver, the oxidation of ethanol to acetaldehyde is mediated by three enzymatic systems: (1) the cytosolic NAD^+^ linked class I alcohol dehydrogenase; (2) the microsomal ethanol oxidizing system; and (3) catalase mainly present in peroxisomes. Catalase oxidizes short-chain primary alcohols by its NAD^+^-independent peroxidatic activity, a pathway potentially important at increased rates of peroxisomal FA β-oxidation [40]. In human fatty liver, peroxisomes were found to increase in both number and density, whereas their size and catalase activity were decreased [41]. A peroxisomal alcohol:NAD^+^ oxidoreductase likely involved in ethanol metabolism has been reported [42]. Furthermore, ADH1A has previously been detected in rat liver peroxisomes [14]. We identified ADH1A as a new candidate protein of human liver peroxisomes. It was found to partially associate with peroxisomes, but not other organelles namely late endosomes/lysosomes and mitochondria (Fig. 3, Fig. S2, S4 and S5). For further functional studies, it would be of great interest to resolve whether this enzyme plays a role in peroxisomal ethanol metabolism and/or has additional functions in human liver peroxisomes.

### Identification of Malate Dehydrogenase 1 and L-lactate Dehydrogenase A Provides New Evidence of NAD^+^ Regeneration by Alternative Shuttle Mechanisms in Human Peroxisomes

β-oxidation of FA is a universal property of peroxisomes shared between different cell types and organisms. In this process, H_2_O_2_ and NADH are generated. The resulting toxic H_2_O_2_ is removed by catalase and regeneration of NAD^+^ by oxidation of NADH takes place via an NAD(H)-redox shuttle mechanism. Since the peroxisomal membrane is generally considered to be impermeable to both NAD^+^ and NADH, the NADH generated during FA β-oxidation has to be reconverted to NAD^+^ inside the organelle; however, the underlying mechanism are still not fully understood for mammalian peroxisomes. Yeast peroxisomes contain a specific malate dehydrogenase that is distinct from the mitochondrial and cytosolic isoenzymes to allow reoxidation of intraperoxisomal NADH [43]. The resultant malate is transported across the peroxisomal membrane and oxidized to oxaloacetate (OAA) by cytosolic MDH. Although MDH1 was identified in mouse kidney peroxisomes [15], it has not yet been considered to localize to mammalian peroxisomes. LDH [44] and enzymes of the glyoxylate cycle [45] were found in rat peroxisomes and a lactate shuttle mechanism was postulated [44], [46].

In this work, we report for the first time a partial association of MDH1 and LDHA with human liver peroxisomes based on subcellular localization studies by quantitative MS and confocal microscopy (Fig. 3, Fig. S2 and Table 1). Furthermore, the recorded activity profiles of MDH and LDH correlated with the activity profile of the peroxisomal marker protein catalase in the dense part of the gradient with highest activity in fraction 6 (Fig. S1). However, as expected both oxidoreductases showed main activities in the lower-density part of the gradient starting from fraction 12. Since the performed activity measurements were not isoform-specific, the measured values presumably reflect the activities of different isoforms of MDH and LDH including mitochondrial LDH and MDH2 in our assay. This generally fits to our observation that LDH and MDH activity profiles exhibited two local maxima along the human liver Nycodenz density gradient in this work (Fig. S1). We conclude from our data that a small, but distinct portion of MDH1 and LDHA localizes to human liver peroxisomes which in turn may point to the existence of alternative shuttle mechanisms underlying the regeneration of NAD^+^ during FA β-oxidation (Fig. S7). We hypothesize that efficient regeneration of NAD^+^ as mediated by MDH1 and/or LDHA is essential to allow human peroxisomes to function optimally at different metabolic states. However, further studies are needed to clarify whether these two enzymes are indeed active constituents of human peroxisomes.

### Concluding Remarks

Our label-free quantitative proteomics survey held the great promise of identifying new proteins associated with human liver peroxisomes. These data are expected to trigger functional studies of newly identified peroxisome-associated proteins and, thus, to advance peroxisome research in general. The partial association of MDH1 and LDHA with human peroxisomes sheds new light on the existence of alternative mechanisms underlying the regeneration of NAD^+^ in this organelle. We further showed that ISOC1 and HSDL2, two proteins with a so far unknown function, localize to peroxisomes. Further analysis of such ill-defined proteins may allow the discovery of new metabolic pathways in peroxisomes. While these data underscore the importance of organellar proteomics studies of human tissues and the high applicability of label-free protein profiling using high resolution MS in general, it also provides a promising route to a better understanding of peroxisomal disorders in humans.

## Supporting Information

Figure S1 **Subcellular distribution of selected marker proteins measured in a Nycodenz gradient of human liver.** A postnuclear supernatant of liver from human tissue was prepared and subjected to equilibrium density-gradient centrifugation as described in the Material and Method section. After centrifugation, the gradient was fractionated into fractions of 2 ml starting from the bottom and marker enzyme activities were measured in all fractions; catalase (peroxisomes), β-hexosaminidase (lysosomes), glutamate dehydrogenase (GlutDH, mitochondria) and esterase (microsomes). In addition, activities of lactate dehydrogenase (LDH) and malate dehydrogenase (MDH) were measured along the density gradient. Results are percentages of total activity observed for each enzyme.
(XLSX)

Figure S2 **Colocalization studies of peroxisomal candidate proteins by confocal microscopy.** In a second, independent experiment, Huh7 cells were transfected with plasmids for expression of ISOC1, HSDL2, MDH1, LDHA or ADH1A each of which fused with its C-terminus to DsRed as well as EGFP-SKL as peroxisomal marker. All peroxisomal candidate proteins showed punctate pattern that corresponded well to the fluorescent pattern of EGFP-SKL. Images from the left to right: EGFP-SKL (green), candidate fusion protein (red), merge. Images were assembled from z-projections; the scale bar represents 20 µm.
(TIF)

Figure S3 **Colocalization studies of the peroxisomal protein PMP52 and DsRed-Mito with EGFP-SKL by confocal microscopy.** Huh7 cells were transfected with plasmids for expression of PMP52 fused with its C-terminus to DsRed as well as DsRed-Mito used as mitochondrial marker. PMP52 colocalized with the peroxisomal marker EGFP-SKL (positive control). Furthermore, the organelle markers EGFP-SKL and DsRed-Mito did not colocalize. Images from the left to right: EGFP-SKL (green), PMP52 fusion protein or Mito marker (red), merge. Images were assembled from z-projections; the scale bar represents 20 µm.
(TIF)

Figure S4 **Colocalization studies of new peroxisomal candidate proteins with late endocytic organelles by confocal microscopy.** Huh7 cells were transfected with plasmids for expression of DsRed fusion proteins of ISOC1, HSDL2, MDH1, LDHA or ADH1A as well as LAMP1-mGFP, a marker for late endosomes and lysosomes. All peroxisomal candidate proteins did not colocalize with LAMP1-mGFP. Images from the left to right: LAMP1-mGFP (green), candidate fusion protein (red), merge. Images were assembled from z-projections; the scale bar represents 20 µm.
(TIF)

Figure S5 **Colocalization studies of new peroxisomal candidate proteins with mitochondria by confocal microscopy.** Huh7 cells were transfected with plasmids for the expression of ISOC1, HSDL2, MDH1, LDHA or ADH1A each of which fused with its C-terminus to DsRed as well as EGFP-Mito used as mitochondrial marker. All peroxisomal candidate proteins did not colocalize with EGFP-Mito with the exception of HSDL2. Additional punctae that did not colocalize with EGFP-Mito were observed for HSDL2 possibly reflecting its partial peroxisomal location. Images from the left to right: EGFP-Mito (green), candidate fusion protein (red), merge. Images were assembled from z-projections; the scale bar represents 20 µm.
(TIF)

Figure S6 **Colocalization studies of HSDL2 and ISOC1 by confocal microscopy.** Huh7 cells were transfected with plasmids for expression of ISOC1 and HSDL2 each of which fused with its N-terminus to DsRed as well as EGFP-SKL as peroxisomal marker. In addition, N-terminally tagged HSDL2 and EGFP-Mito were expressed. Both fusion proteins showed a punctate pattern that corresponded well to the fluorescent pattern of EGFP-SKL. A fraction of each protein was also observed in the cytosol. In addition, DsRed-HSDL2 did not colocalize with the mitochondrial marker EGFP-Mito (lower row). Images from the left to right: EGFP-SKL or EGFP-Mito (green), candidate fusion protein (red), merge. Images were assembled from z-projections; the scale bar represents 20 µm.
(TIF)

Figure S7 **Schematic representation of peroxisomal β-oxidation of fatty acids and alternative shuttle mechanisms proposed for the regeneration of NAD^+^ in human peroxisomes.** Fatty acids are degraded by β-oxidation yielding acetyl-CoA and the respective acyl-CoA shortened by two carbon atoms per cycle. In human, the four reactions including: (1.) oxidation; (2.) hydration; (3.); dehydrogenation and (4.) thiolysis per cycle are carried out by multiple enzymes as depicted; abbreviations are ACOX, acyl-CoA oxidase; DBP, D-bifunctional enzyme; LBP, L-bifunctional enzyme and SCPx, sterol carrier protein X. Since the peroxisomal membrane is apparently impermeable to NAD^+^/NADH, regeneration of NAD^+^ has to take place inside peroxisomes to allow continued fatty acid β-oxidation. The partial association of both LDHA and MDH1 with human peroxisomes may point to the existence of alternative shuttle mechanisms for the efficient regeneration of NAD^+^. The substrates and products of MDH1 (malate and oxaloacetate) and LDHA (lactate and pyruvate) are presumably shuttled across the peroxisomal membrane thereby regenerating NAD^+^ to meet the demands of fatty acid β-oxidation in human peroxisomes.
(TIF)

Table S1 **List of all proteins identified by MS-based proteomic analyses of the peroxisomal peak fraction (fraction 6) of the Nycodenz density gradient.**
(XLSX)

Table S2 **List of all proteins identified by MS-based proteomic analyses of Nycodenz density gradient fractions 4 to 12 of human liver.** Additional information about peptide identifications is listed in Table S4. For full spectral information visit www.ebi.ac.uk/PRIDE with accession 11791.
(XLSX)

Table S3 **Catalogue of known peroxisomal proteins identified in human liver peroxisomes.**
(XLSX)

Table S4 **Average of normalized peptide abundance in fractions 4 to 12 of the density gradient and the Euclidian distance calculated for each protein.** The first 46 proteins (peroxisomal) were used to calculate the mean peroxisomal profile (for further details please refer to Methods). Proteins confirmed as new peroxisomal proteins in human liver in this work are marked with an asterisk (*).
(XLSX)

Table S5 **List of all proteins identified by MS-based proteomic analyses of Nycodenz density gradient fractions 4 to 12 of human liver.** Information about peptide identifications are marked in italics.
(XLSX)

Table S6 **Peptide abundances as calculated by DeCyder MS 2.0.** Intensities given are log_2_ values of peptide intensities.
(XLSX)

Table S7 **Primers used in this study with restriction sites underlined.**
(DOCX)

Text S1 **Molecular Cloning.**
(DOCX)
