# Correction: The Proteome of Human Liver Peroxisomes: Identification of Five New Peroxisomal Constituents by a Label-Free Quantitative Proteomics Survey

**DOI:** 10.1371/annotation/3552e5c7-88d1-42c5-844d-4c2f2d722533

**Published:** 2013-10-16

**Authors:** Thomas Gronemeyer, Sebastian Wiese, Rob Ofman, Christian Bunse, Magdalena Pawlas, Heiko Hayen, Martin Eisenacher, Christian Stephan, Helmut E. Meyer, Hans R. Waterham, Ralf Erdmann, Ronald J. Wanders, Bettina Warscheid

An error in the file extension was introduced during the production process for Figure S1. Figure S1 can be viewed here: 
