# Supplementary figures and images for: The Proteome of Human Liver Peroxisomes: Identification of Five New Peroxisomal Constituents by a Label-Free Quantitative Proteomics Survey
Source: PLoS One. 2013 Feb 27;8(2):e57395. doi: 10.1371/journal.pone.0057395 (PMC3583843; doi:10.1371/journal.pone.0057395)

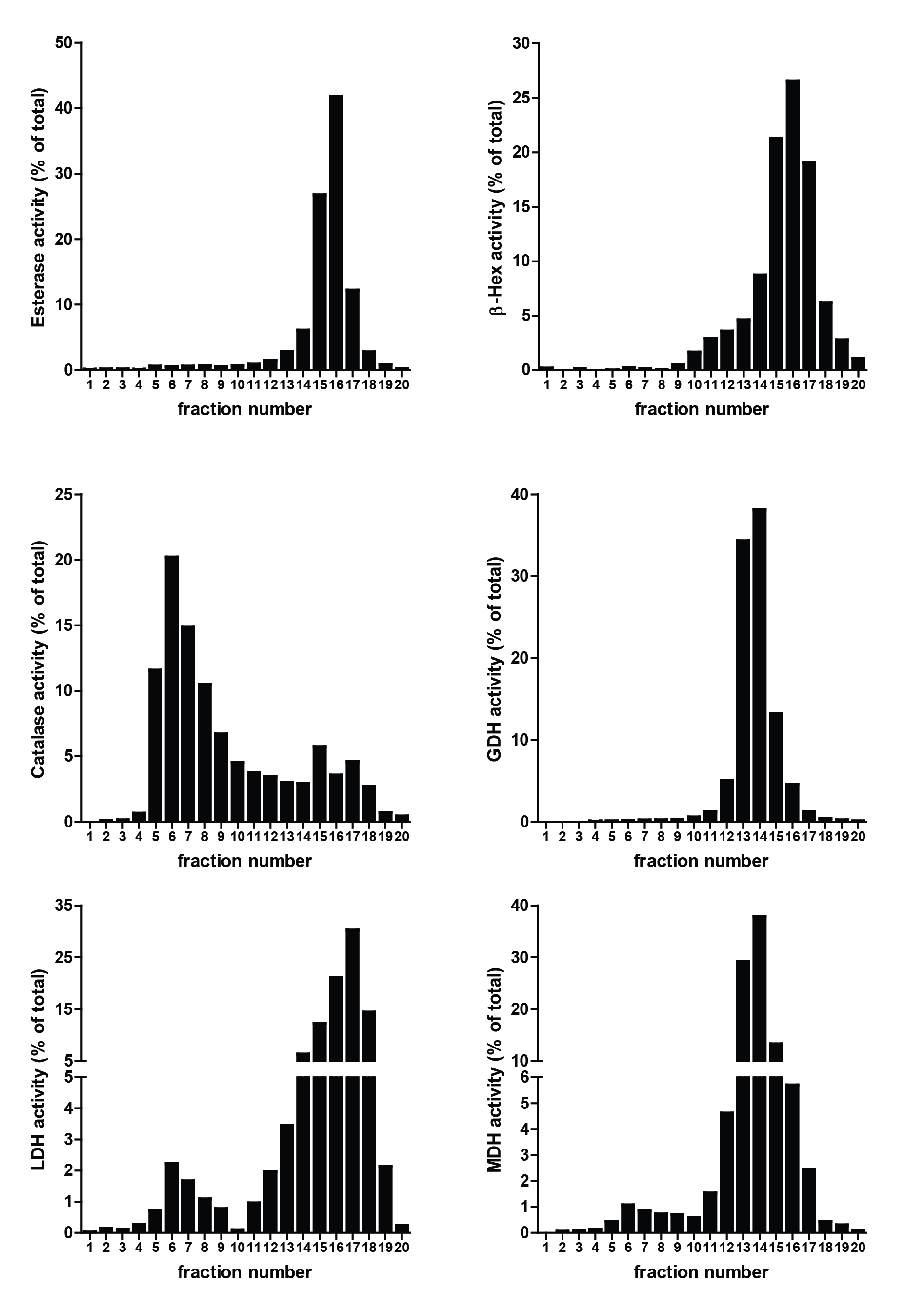

Supplement: Figure S1 — Subcellular distribution of selected marker proteins measured in a Nycodenz gradient of human liver. A postnuclear supernatant of liver from human tissue was prepared and subjected to equilibrium density-gradient centrifugation as described in the Material and Method section. After centrifugation, the gradient was fractionated into fractions of 2 ml starting from the bottom and marker enzyme activities were measured in all fractions; catalase (peroxisomes), β-hexosaminidase (lysosomes), glutamate dehydrogenase (GlutDH, mitochondria) and esterase (microsomes). In addition, activities of lactate dehydrogenase (LDH) and malate dehydrogenase (MDH) were measured along the density gradient. Results are percentages of total activity observed for each enzyme. (XLSX) [file pone.0057395.s001.xlsx]

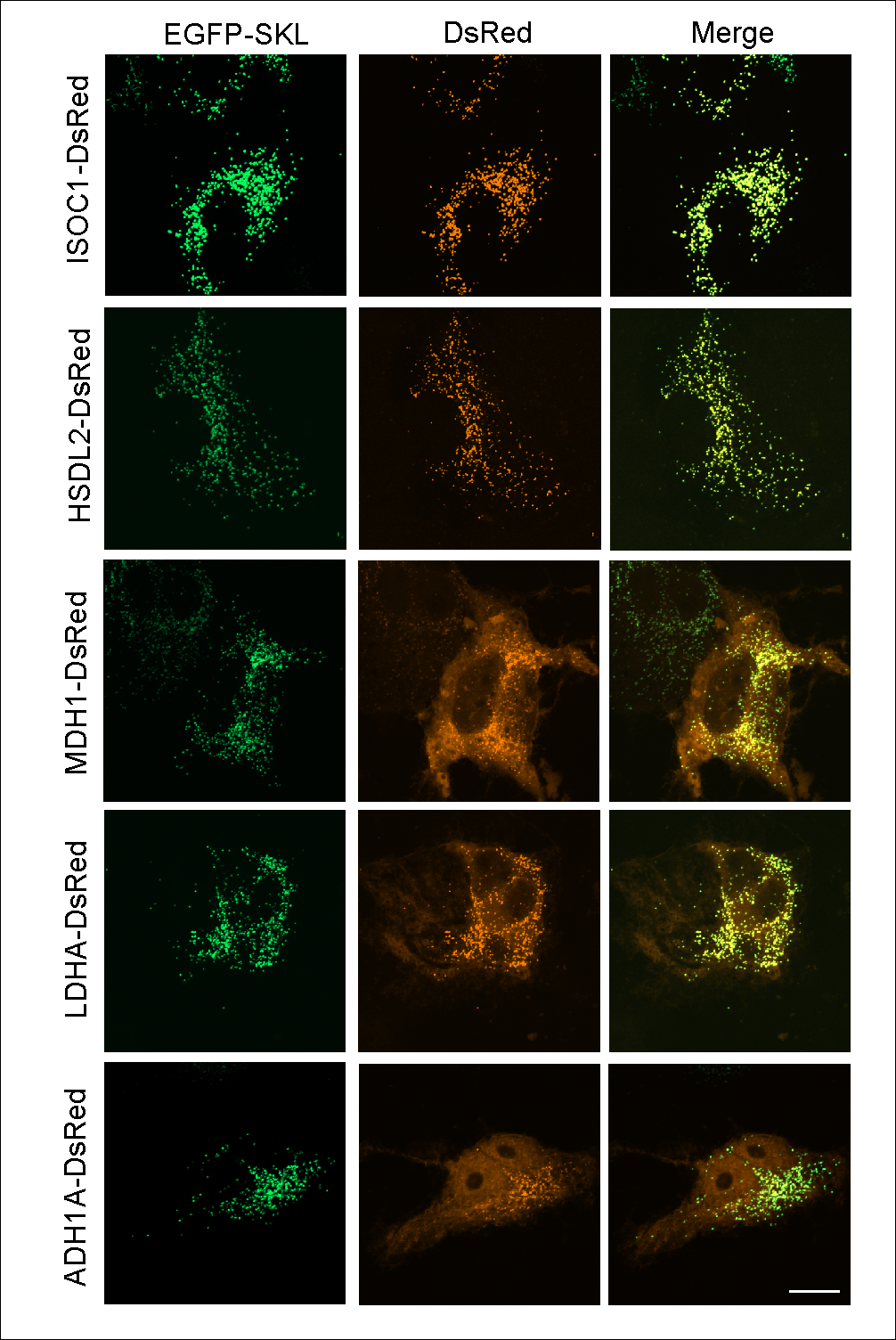

Supplement: Figure S2 — Colocalization studies of peroxisomal candidate proteins by confocal microscopy. In a second, independent experiment, Huh7 cells were transfected with plasmids for expression of ISOC1, HSDL2, MDH1, LDHA or ADH1A each of which fused with its C-terminus to DsRed as well as EGFP-SKL as peroxisomal marker. All peroxisomal candidate proteins showed punctate pattern that corresponded well to the fluorescent pattern of EGFP-SKL. Images from the left to right: EGFP-SKL (green), candidate fusion protein (red), merge. Images were assembled from z-projections; the scale bar represents 20 µm. (TIF) [file pone.0057395.s002.tif]

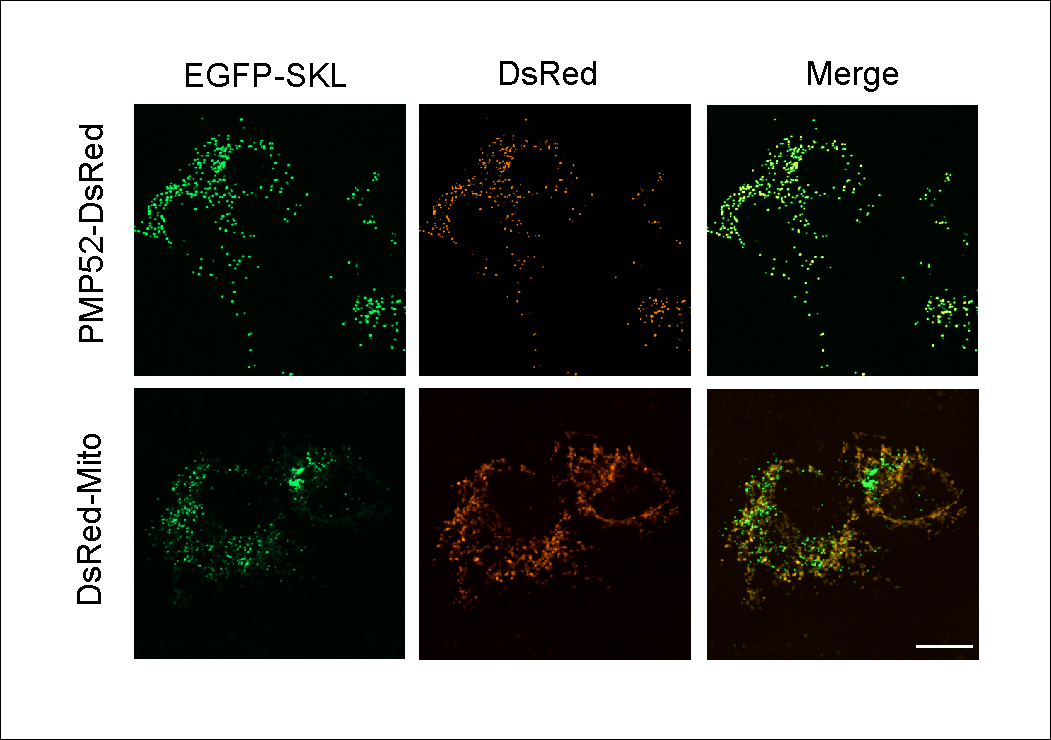

Supplement: Figure S3 — Colocalization studies of the peroxisomal protein PMP52 and DsRed-Mito with EGFP-SKL by confocal microscopy. Huh7 cells were transfected with plasmids for expression of PMP52 fused with its C-terminus to DsRed as well as DsRed-Mito used as mitochondrial marker. PMP52 colocalized with the peroxisomal marker EGFP-SKL (positive control). Furthermore, the organelle markers EGFP-SKL and DsRed-Mito did not colocalize. Images from the left to right: EGFP-SKL (green), PMP52 fusion protein or Mito marker (red), merge. Images were assembled from z-projections; the scale bar represents 20 µm. (TIF) [file pone.0057395.s003.tif]

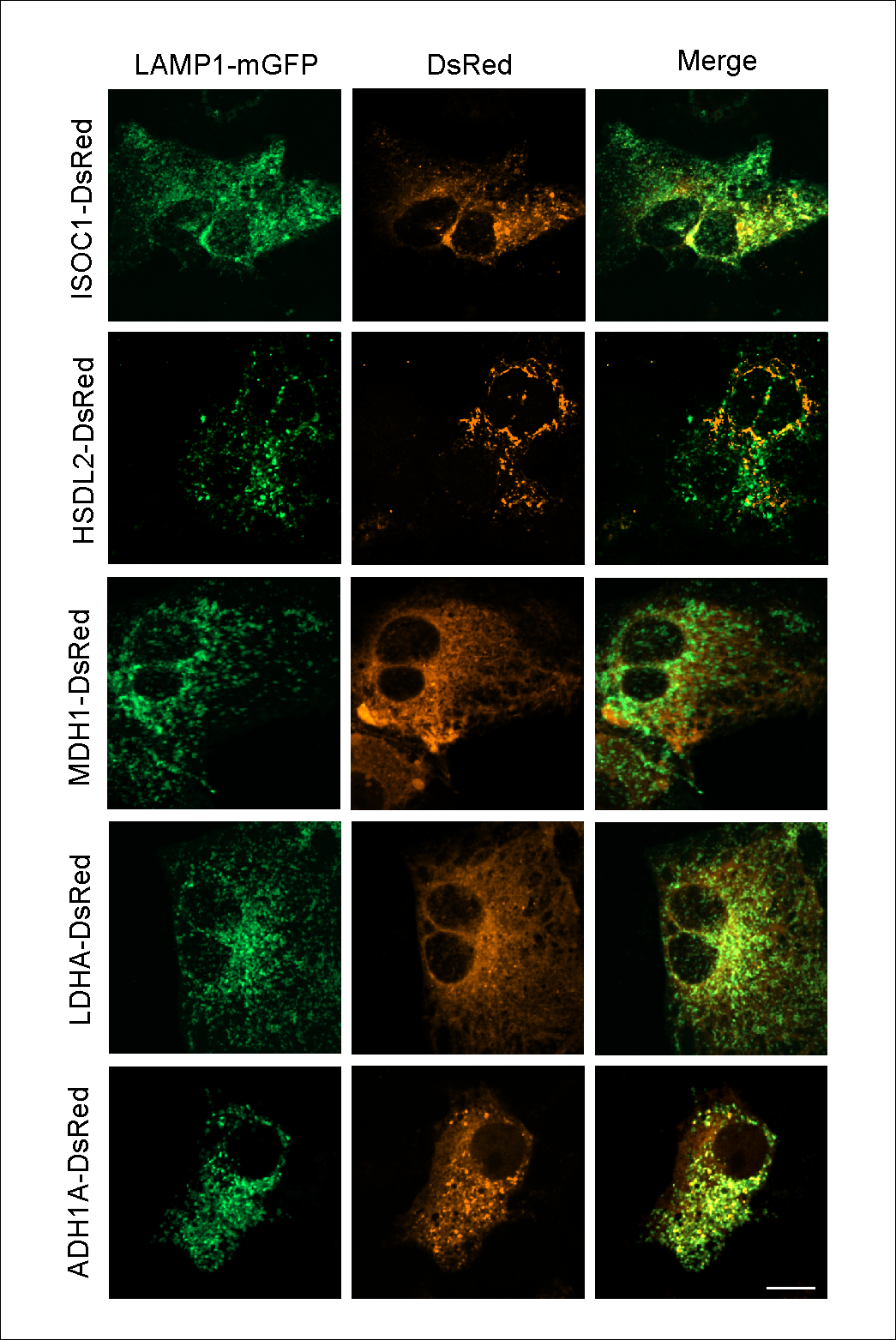

Supplement: Figure S4 — Colocalization studies of new peroxisomal candidate proteins with late endocytic organelles by confocal microscopy. Huh7 cells were transfected with plasmids for expression of DsRed fusion proteins of ISOC1, HSDL2, MDH1, LDHA or ADH1A as well as LAMP1-mGFP, a marker for late endosomes and lysosomes. All peroxisomal candidate proteins did not colocalize with LAMP1-mGFP. Images from the left to right: LAMP1-mGFP (green), candidate fusion protein (red), merge. Images were assembled from z-projections; the scale bar represents 20 µm. (TIF) [file pone.0057395.s004.tif]

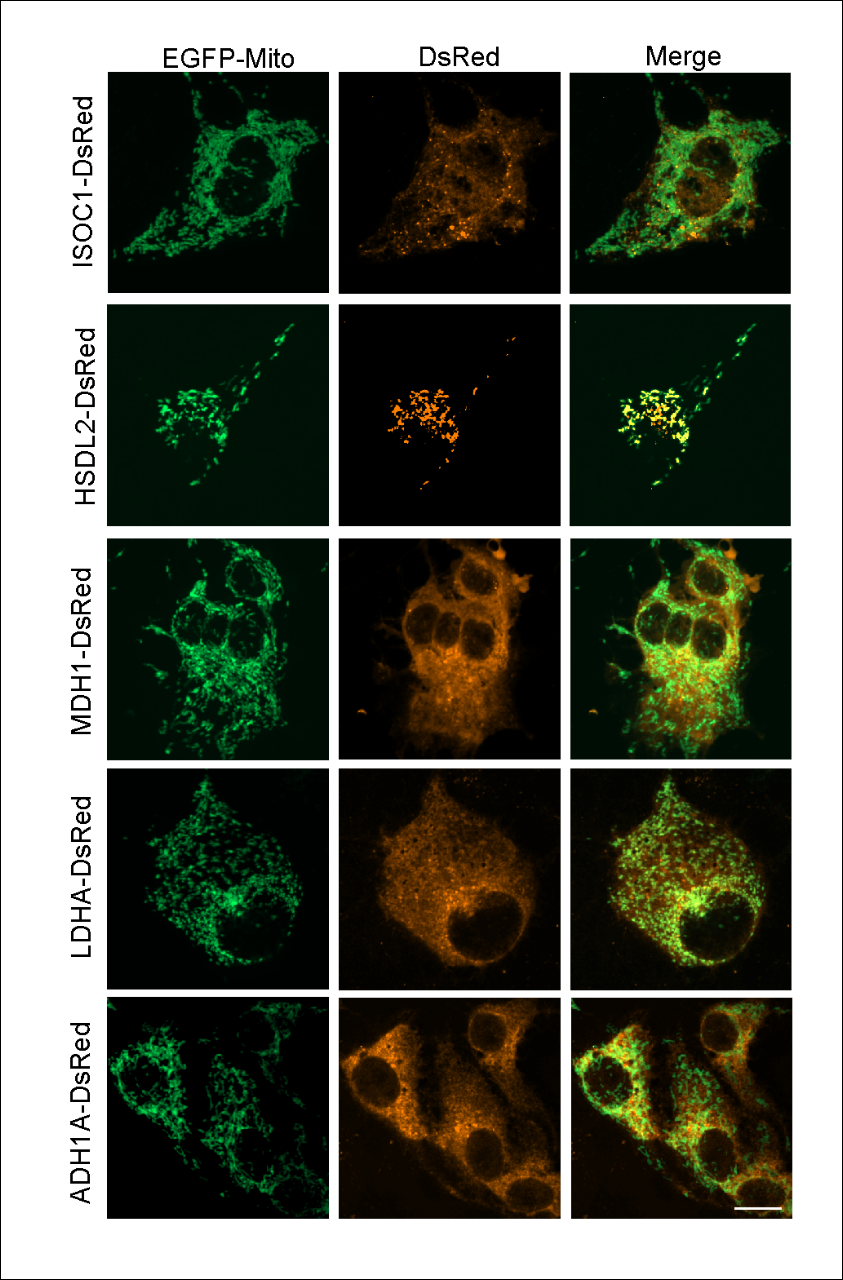

Supplement: Figure S5 — Colocalization studies of new peroxisomal candidate proteins with mitochondria by confocal microscopy. Huh7 cells were transfected with plasmids for the expression of ISOC1, HSDL2, MDH1, LDHA or ADH1A each of which fused with its C-terminus to DsRed as well as EGFP-Mito used as mitochondrial marker. All peroxisomal candidate proteins did not colocalize with EGFP-Mito with the exception of HSDL2. Additional punctae that did not colocalize with EGFP-Mito were observed for HSDL2 possibly reflecting its partial peroxisomal location. Images from the left to right: EGFP-Mito (green), candidate fusion protein (red), merge. Images were assembled from z-projections; the scale bar represents 20 µm. (TIF) [file pone.0057395.s005.tif]

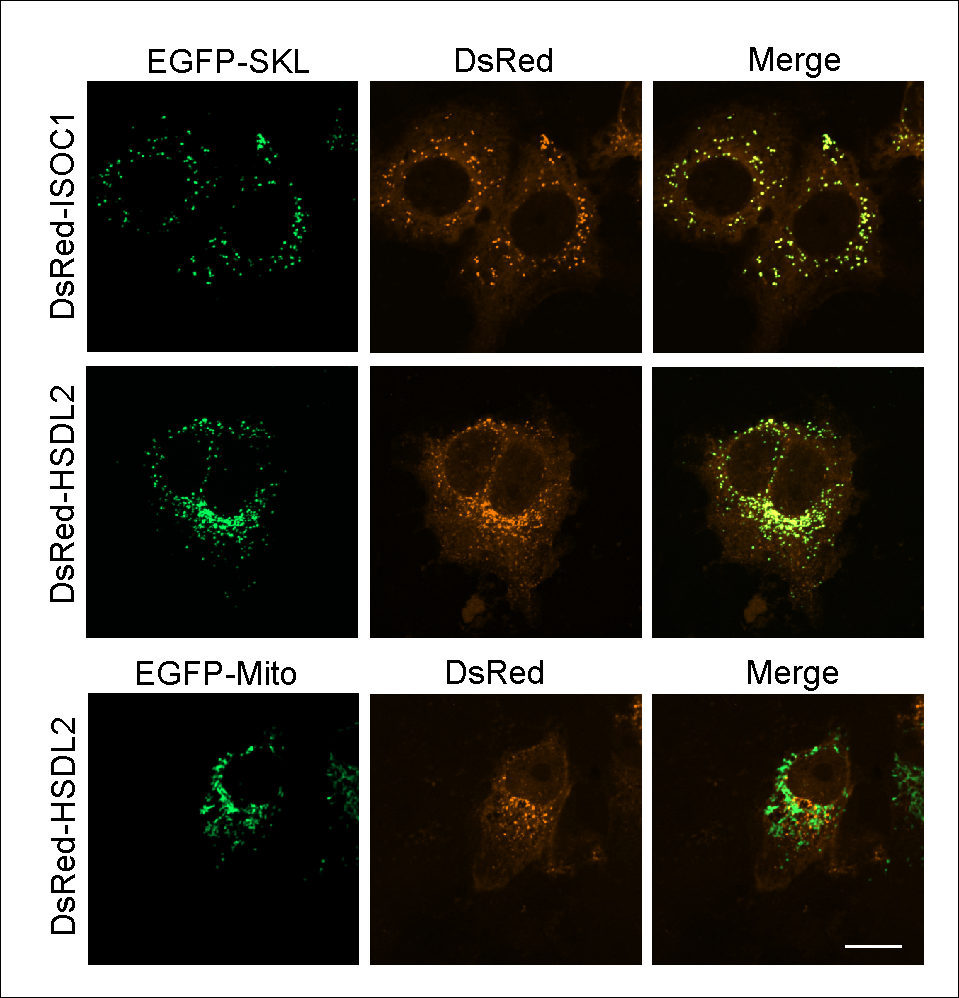

Supplement: Figure S6 — Colocalization studies of HSDL2 and ISOC1 by confocal microscopy. Huh7 cells were transfected with plasmids for expression of ISOC1 and HSDL2 each of which fused with its N-terminus to DsRed as well as EGFP-SKL as peroxisomal marker. In addition, N-terminally tagged HSDL2 and EGFP-Mito were expressed. Both fusion proteins showed a punctate pattern that corresponded well to the fluorescent pattern of EGFP-SKL. A fraction of each protein was also observed in the cytosol. In addition, DsRed-HSDL2 did not colocalize with the mitochondrial marker EGFP-Mito (lower row). Images from the left to right: EGFP-SKL or EGFP-Mito (green), candidate fusion protein (red), merge. Images were assembled from z-projections; the scale bar represents 20 µm. (TIF) [file pone.0057395.s006.tif]
