# Supplementary material for: The Proteome of Human Liver Peroxisomes: Identification of Five New Peroxisomal Constituents by a Label-Free Quantitative Proteomics Survey
Source: PLoS One. 2013 Feb 27;8(2):e57395. doi: 10.1371/journal.pone.0057395 (PMC3583843; doi:10.1371/journal.pone.0057395)
Supplement: Table S7 — Primers used in this study with restriction sites underlined. (DOCX) [file pone.0057395.s014.docx]

**Table S2. Primers used in this study with restriction sites underlined.**

| **Primer name** | **Sequence 5’→3’** |
| --- | --- |
| PMP52-Nhefor | GATCGAGCTAGCG GTC GCC ACC AT*G* GCG GCC CTC AGC AAG TC |
| PMP52-back | GCATG AC CGG TAG GGA AAA CTC TGT GGG CAA CTC |
| ISOC-Sacfor | GCAACGA GCT CAA TAC CCA TAC GAT GTT CCA GAT TAC GCT ATG  GCG GCT GCG GAG CCG GC |
| ISOC-Xmaback | CGGAT CCCGGGCC TTA TAC TTT GGA AAG CAG ACC |
| ISOC-Nhefor | GATCGAGCTAGCG GTC GCC ACC ATG GCG GCT GCG GAG CCG |
| ISOC-Ageback | GCATG AC CGG TAG TAC TTT GGA AAG CAG ACC |
| HSDL-Nhefor | GATCGAGCTAGCG GTC GCC ACC ATG TTACCCAACACCGGG |
| HSDL-Ageback | GCATGACCGGTAGCAGTCTGGCATTCATCTGATTC |
| HSDL-Sacfor | GCAACGA GCT CAA ATG TTACCCAACACCGGG |
| HSDL-Xmaback | CGGATCCCGGGCCTTACAGTCTGGCATTCATCTGATTC |
| LACT-Nhefor | GATCGAGCTAGCG GTC GCC ACC ATG GCTGCTGTACTGCAGCG |
| LACT-Ageback | GCATGACCGGTAGAAGATGAGCTTTCCATTTCTTGTC |
| MDH-Nhefor | GATCGAGCTAGCG GTC GCC ACC ATG TCT GAA CCA ATC AGA GTC C |
| MDH-Ageback | GCATGACCGGTAGGGCAGAGGAAAGAAATTCAAAAGC |
| LDHA-Nhefor | GATCGAGCTAGCG GTC *GCC ACC* ATGGCAACTCTAAAGGATCAGC |
| LDHA-Ageback | GCATGACCGGTAGAAATTGCAGCTCCTTTTGGATC |
| ADH1-Nhefor | ATCGAGCTAGCG GTC GCC ACC ATGAGCACAGCAGGAAAAGTAATC |
| ADH1-Ageback | GCATGACCGGTAGAAACATCAGAATGGTACGGATAC |
