# Supplementary material for: The Proteome of Human Liver Peroxisomes: Identification of Five New Peroxisomal Constituents by a Label-Free Quantitative Proteomics Survey
Source: PLoS One. 2013 Feb 27;8(2):e57395. doi: 10.1371/journal.pone.0057395 (PMC3583843; doi:10.1371/journal.pone.0057395)
Supplement: Text S1 — Molecular Cloning. (DOCX) [file pone.0057395.s015.docx]

**Text S1. Molecular Cloning.**

cDNA clones were purchased from Imagene (Berlin, Germany). The ORFs of the candidate genes were amplified from cDNA clones by PCR and subsequently cloned into the eukaryotic expression vector pDsRed-C1-monomer (BD Biosciences/Clontech, Heidelberg, Germany) using the *Nhe1*/*Age1* or *Sac1*/*Xma1* restriction sites, leading to N- or C-terminal fusion with the red fluorescent protein DsRed. Correct insertion of the insert was confirmed by sequencing of each clone. Table S7 provides information about primers used for cloning.
