# Supplementary figures and images for: Correction: The Proteome of Human Liver Peroxisomes: Identification of Five New Peroxisomal Constituents by a Label-Free Quantitative Proteomics Survey
Source: PLoS One. 2013 Oct 16;8(10):10.1371/annotation/3552e5c7-88d1-42c5-844d-4c2f2d722533. doi: 10.1371/annotation/3552e5c7-88d1-42c5-844d-4c2f2d722533 (PMC3812376; doi:10.1371/annotation/3552e5c7-88d1-42c5-844d-4c2f2d722533)

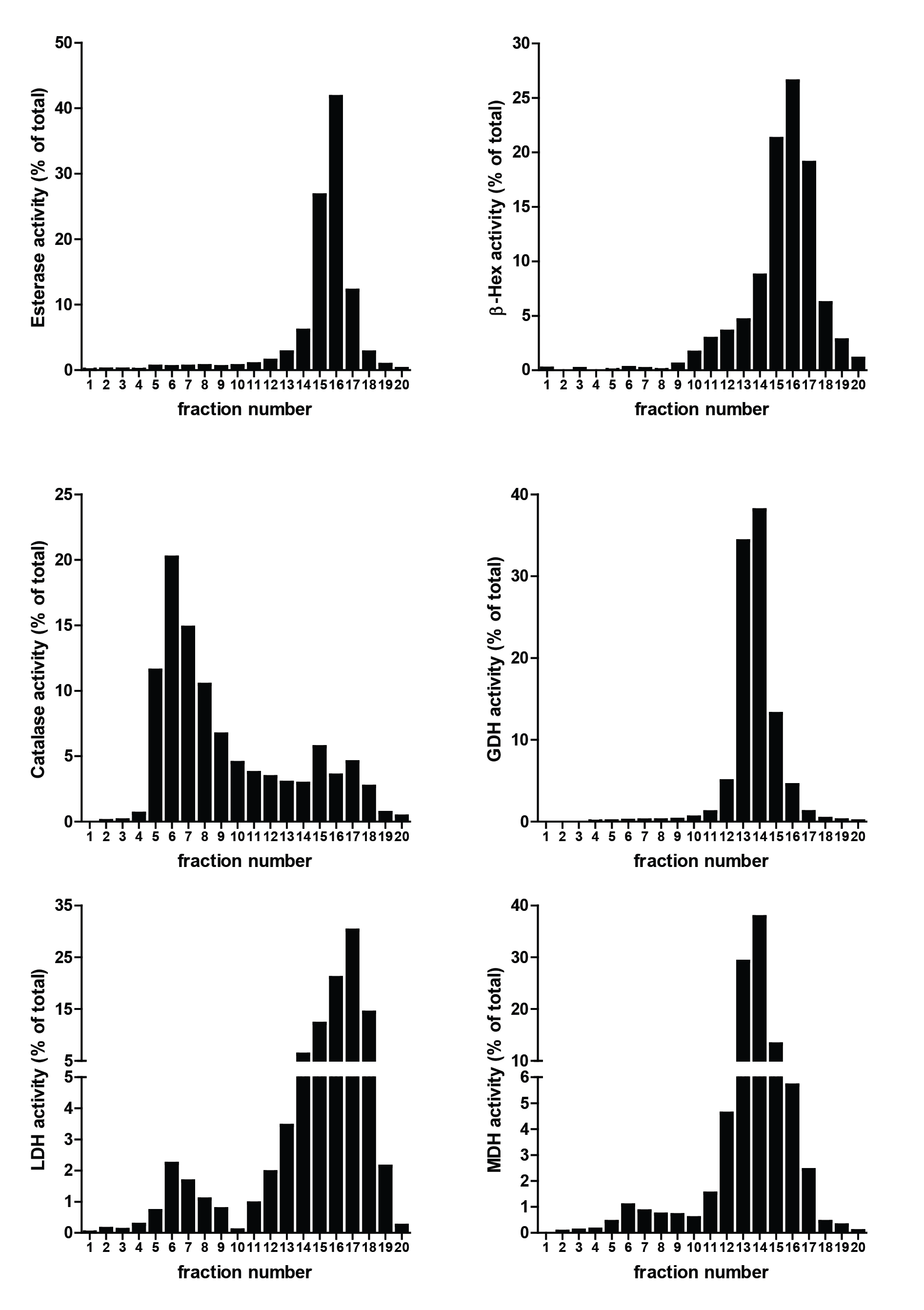

Supplement: Supplementary file 1 [file pone.3552e5c7-88d1-42c5-844d-4c2f2d722533.s001.tif]
